# Supplementary material for: Are carbon emissions trading and green financial instruments synergistic? -Comprehensive quantitative research based on content analysis
Source: PLoS One. 2024 Mar 7;19(3):e0298601. doi: 10.1371/journal.pone.0298601 (PMC10919601; doi:10.1371/journal.pone.0298601)
Supplement: S1 Data — (ZIP) [file pone.0298601.s001.zip › Data/地级市Z_F工作报告.zip!/╜¡╦╒/┴1⁄4╘╞╕█╩╨/└┤╫╘╣1⁄2╓┌║┼í╛┬φ┐╦╩2╛▌═°í┐_2005.docx]

现在，我代表市人民政府向大会作工作报告，请予审议，并请各位政协委员提出意见。

2004年工作回顾

2004年，全市人民在中共连云港市委的领导下，以邓小平理论和三个代表重要思想为指导，认真贯彻党的十六大和十六届三中、四中全会精神，树立和落实科学发展观，立足加快发展，突出真抓实干，加快建设以人为本、全面协调可持续发展的新港城，顺利完成了市十一届人大二次会议确定的各项任务。

一、经济快速健康发展

认真落实国家宏观调控政策，推动经济快速健康发展。实现地区生产总值416.4亿元，增长13.9％，创七年来最高增速，三次产业结构调整为21.3∶45.2∶33.5。人均地区生产总值突破1000美元，经济发展迈上新的台阶。完成财政总收入42.33亿元，增长31.4％，其中一般预算收入18.58亿元，同口径增长35.5％，增幅超过全省平均水平。完成全社会固定资产投资246.3亿元，增长16％。实现社会消费品零售总额141.2亿元，增长14.2％。中心城市综合实力全国百强排名从2001年83位提升到72位。

农村经济加快发展，实现粮食增产农业增效。粮食总产达260.3万吨，增长27.9％。农业总产值达165.9亿元，增长7.7％。预计农民人均纯收入3604元，增长14.8％。农村劳务输出百万工程有序推进，新增劳务输出14.4万人。农业标准化步伐加快，40个产品获国家无公害品牌证书，无公害、绿色和有机食品品牌达到87个，四县全部成为无公害农产品整体推进县。建成十大农产品出口创汇基地，农产品出口创汇达2.3亿美元，连续第五年保持全省领先。渔业经济向规模化、特色化、精品化发展，海淡水放养面积97.1万亩，工厂化养殖突破20万平方米，海洋捕捞创十年来最好水平，青口港升格为国家级中心渔港，渔业资源修复工程初见成效。有效预防禽流感疫情，初步构建畜禽良种化和产业化经营体系，畜牧业成为农民增收的重要增长点。实施云台山封山育林、城郊观光林业等八项重点工程，林业产业化水平不断提高，东海县和淮海工学院被评为全国绿化模范单位。年销售超亿元龙头企业达18家，兴云集团跻身国家级龙头企业行列。农机跨区作业连续第六年被评为全国先进。小塔山水库除险加固、烧香河北闸拆建、海堤达标等一批重点水利工程顺利推进。

工业经济快速发展，工业强市方略扎实推进。坚持走新型工业化道路、提升工业主体地位，实现工业增加值144亿元，增长18.6％，占地区生产总值比重提高2.1个百分点。规模以上工业实现销售收入261.2亿元，增长37.6％，实现利税25.9亿元，增长23.3％。临港开发深入实施，沿东陇海线产业带加快建设，浮法玻璃一期、益海粮油二期等项目建成投产，东部城区规模以上工业产值增幅高于全市12个百分点。工业结构进一步优化，发展活力不断增强，股份制、外资、私营工业增幅均超过30％，80个新增长点项目拉动工业销售收入增长18个百分点，36户高新技术企业产值增长35.2％，信息产品制造业实现产值12亿元。工业经济加速集聚发展，骨干行业拉动作用增强，新增工业产值70％以上来自食品、化工、医药三大行业，新开工过千万元的235个工业项目80％以上进入园区。规模企业培育成效明显，新增规模以上工业企业161家。完成工业投资138.1亿元，25个重点项目竣工投产，40个重点项目先后开工。核电站1、2号机组安装调试，伊芦、新滩等一批输变电及配套工程顺利实施。建筑业实现增加值26.2亿元，带动劳务输出18.8万人。

服务业繁荣发展，新型服务业发展格局开始形成。商贸流通向商业中心区、新型流通业态、流通骨干企业集中发展，一批著名商业品牌进入连云港市场，20家重点流通企业实现销售40亿元。居民消费价格指数102.9。孔望山景区被评为国家4A级旅游区，港口、宋口村、振兴花卉园被评为全国首批工农业旅游示范点，成功举办连云港之夏、花果山世界儿童联欢节等旅游节庆活动。接待国内外游客增长21.8％，实现旅游收入增长23％。数字连云港建设加快，电子政务、电子商务、企业信息化快速发展，新增宽带用户4万户、信息化小区21个，电话交换机容量达到229万门，杰瑞软件开发、无线电技术设施建设和港口公共基础信息平台项目快速推进。开发房地产270万平方米，完成商品房销售额27.3亿元，增长58.3％。小区物业管理面积增加87万平方米，省级物业管理优秀项目达18个。信贷有效投放得到加强，银企合作进一步改善，金融机构存贷款余额分别比年初增加66.2亿元和41.4亿元，保费收入增长12.3％。

港口经济开拓振兴，民营经济不断壮大，县域经济快速推进，成为经济发展重要增长极。港口功能进一步提升，建成10万吨级散货和1个第五代集装箱泊位，完成7万吨级航道扩建。港口实现吞吐量4352万吨，增长16％，集装箱运量50.2万标箱，增长66.8％，高于全国平均增幅40.4个百分点。以港口为中心的区域性国际物流中心建设全面展开，开通至美西、地中海海上航线和韩国客货班轮航线，开通至阿拉木图集装箱班列，中云物流园区部分建成使用，建成全国首家专业棉花保税库。集疏运条件进一步改善，连霍国道主干线全线通车，连盐高速公路加快建设，部分国省道得到改造，公路运输建成卫星定位系统。东陇海铁路电气化改造列入国家十一五计划。民航候机楼投入使用，航线航班密度大幅增加。大力推动全民创业，建立民营经济发展服务机构，充实完善信用担保体系，民营经济发展环境进一步优化。企业登记注册户数和注册资金分别增长22.2％和54.2％，新增从业人员3.5万人，出现一批规模较大的民营企业。民营经济实现增加值186.6亿元，占地区生产总值44.8％，对全市经济增长贡献率超过60％。县域经济统筹发展，工业立县成效明显，海洋资源、硅资源、农副产品资源开发各具特色，呈现产业化推进、集聚化发展的良好势头。县域全社会固定资产投资增幅比全市高出22.7个百分点，四县外商直接投资占全市比重达54.1％，提高18.5个百分点，新开工1000万元以上工业项目占全市66.4％。四县财政收入增幅全部进入苏北前十名行列。

二、改革开放持续推进

继续深化各项改革，市场体制日趋完善。加快投融资体制改革，吸引中海集团等投资主体加快港口建设，中金药包、德邦集团实现外资参股。豪森药业等4家企业完成上市辅导。有效重组存量资产3.6亿元。新组建水利、教育和新浦3家资产经营公司，县区基本建立国有资产经营管理体系，国有资本运作能力增强。积极推进财政国库管理制度改革，组建市国库集中支付中心。启动事业单位改革，首批67家生产经营型单位改制转企。新发展为农服务社100家，2家合作社被评为国家示范专业合作社。

强化开放载体建设，园区集聚效应不断增强。国家级开发区基础设施投入超过前五年总和，进区项目平均投资规模突破1000万美元。临港产业区12平方公里起步区具备项目进区条件，出口加工区、宋跳高新区项目布局基本完成，大浦工业区加快建设。县区开发园区快速发展，4个省级和6个拟保留园区基础设施建设投入6.3亿元。12个开发园区每平方公里项目投资6亿元，每平方公里业务总收入11.2亿元，分别提高50％和20％，园区经济成为区域经济的重要增长极和招商引资的主要载体。成功承办第六届江苏农业国际合作洽谈会。外商直接投资3.01亿美元，增长43.3％，内资客方到位资金75亿元，实现倍增目标。

外贸外经快速增长。完成外贸进出口总额15.4亿美元，增长61.7％，其中出口7.6亿美元，增长31.8％。重点项目、重点企业支撑作用加强，外商投资企业和出口生产企业出口比重明显提高，10家重点企业完成出口占全市54％。出口商品结构优化，出口市场不断扩大，高新技术产品比重占15.3％，本地产品比重达64.8％。完成外经营业额1.3亿美元，增长14.8％，年末在外劳务人员1.2万人。积极开展对外投资，康缘在香港成立实业公司，蓝天集团在马来西亚开发锡矿项目。海关、检验检疫、海事、边检在通关、预检等方面创新服务形式，大通关、大物流环境进一步优化。

三、城乡建设明显加快

扩大城市规划覆盖率，城市总体规划成果得到完善，四县县城和港口总体规划修编完成，采用国内外招标方式组织编制连岛总体规划，采用专家评审方式组织编制开发区发展概念规划和新华广场、凤凰新城等重点地段规划，精心组织编制新浦、海州分区规划和临港产业区、城市出入口等20多项控制性、修建性规划。

中心城市功能进一步完善，城市形象明显提升。投资40.2亿元实施390项城市建设项目，建成区面积扩大到65平方公里。新增城市道路38.6公里，新建改建海连东路、解放西路等41条城市主次干道，新海城区一环、二环路网基本形成。新区建设初具规模，路网管线等市政设施基本完成，市行政中心主楼竣工，住宅开发成片推进。东部城区建设全面展开，新港城大道进展顺利，新东方大道开工建设，开发区至渔湾、板桥两条城郊公路完成拓宽改造，国际商务中心开工建设，建成海滨大道亮化工程、开发区防洪保安工程。公共设施建设加快，建成大浦污水处理厂二期工程，新海城区达到10万吨污水二级处理能力，市危险废弃物集中处置中心投入使用，东盐河和玉带河拓宽整治工程进展顺利。建成人防工程4万平方米，人防应急指挥中心开工建设。有效化解拆迁矛盾，城市建设顺利推进。新增城市亮化面积160万平方米。成立行政执法局，强化综合执法，加强定点管理和机动巡查，市容环境卫生进一步改善。

四县城区投资31亿元实施234项建设项目，新区开发、旧城改造同步实施，道路、公园、广场等一批市政公用设施和标志性工程相继建成。省新型示范小城镇和重点中心镇发挥特色优势，提高规划建设档次，海头、温泉、板浦、汤沟等10个重点中心镇快速发展。市域城镇体系不断完善，城镇化率提高1.8个百分点。

四、社会事业协调发展

科技教育健康发展。组织实施省以上火炬计划项目19个、星火计划项目64个，2个项目列入省成果转化资金重大专项。新增省以上高新技术企业2家，8个产品被认定为国家级新产品、16个产品成为省高新技术产品，6家民营企业列入省首批百家重点民营科技企业培育计划，全国农村星火富民经验交流会在我市召开。新医药产业基地、硅材料产业基地分别被认定为省首批高新技术特色产业示范基地和国家火炬计划特色产业基地，东海县列入国家粮食丰产科技工程核心试验区，中电华威、正大天晴被国家列为博士后科研工作站。位于东海县境内的大陆科钻一井进尺突破5000米，在我国钻探技术史上建立了一座新的里程碑。产学研联合深入推进，一批科技合作项目取得重要进展，百名教授科技兴百村和百名专家科技兴百企工程取得成效。一批哲学社会科学研究成果获省优秀奖。基础教育继续加强，基本完成中小学布局调整，新扩建中小学校舍19.1万平方米，创建一批省级示范幼儿园、实验小学和示范初中，新海高中东方分校正式挂牌，3所学校通过国家级重点职业中学验收，高中段入学率达82.9％。普通高校新生录取率提高5个百分点，地方高校专业和重点实验室建设得到加强。职业技术教育和社会教育加快发展，中药学校、财经学校升格为高等职业技术学校，社会办学规范有序。校园周围环境和网吧专项整治成效明显。

社会事业协调推进。广场文化系列活动蓬勃开展，建成一批省文化网共享工程基层网点和文化先进村，女子民乐团参加中法文化年交流活动，一批作品获得省五个一工程奖，文化市场和新闻出版管理得到加强。广播电视初步实现节目采编数字化，有线电视村联网率达50％。成功承办世界男子和亚洲沙滩排球、全国武术散打等重大赛事，获省级以上竞赛奖牌122枚，其中金牌41枚。全民健身运动蓬勃发展，新建健身工程和网点33个。推荐美猴王为北京奥运会吉祥物工作有序推进。市一院东方分院挂牌运作，社区卫生服务规范发展，临床用血90％以上来自无偿献血，公共卫生体系建设进一步加强，传染病发病率低于全省平均水平。一批社会事业建设项目快速推进，新中波发射台、广电中心二期工程、新海高中艺体馆建成使用。国防教育得到加强，‘淮海一号’演习取得圆满成功，民兵、预备役高炮分队实弹射击比武名列全省前茅，在全省率先建立市级拥军协会。建成省级防震减灾科普教育基地。统计信息功能进一步发挥，完成经济普查单位清查。外事、侨务、对台等工作有效服务经济建设，档案、地方志、民族宗教、关心下一代等工作不断推进。

可持续发展得到加强。人口与计划生育工作稳步推进，基层基础工作进一步规范，生殖健康促进工程和关爱女孩行动工程加快实施，人口自然增长率控制在5.5‰，计划生育率达90％。加强国土资源保护，整治土地市场秩序，复垦土地1万亩，全面关停市区非法采石塘口，注重废弃塘口植被恢复。强化海洋综合管理，海洋执法迈入全国先进行列。加快创建环保模范城市，生态环境建设全面推进，市区空气质量优良率88.5％，蔷薇河饮用水源水质达标率100％，进入全国污染控制和道路声环境质量最佳城市行列。

五、人民生活不断改善

注重改善民生，城乡居民收入水平和生活质量不断提高。全面小康社会建设按照预定目标扎实推进。城市居民人均可支配收入8872元，增长14％。努力扩大就业岗位，新增就业2.48万人，城镇登记失业率4.3％。实施再就业援助，使2.52万名下岗困难职工再就业，1377名4050人员通过政府购买岗位实现再就业。破产关闭企业1.3万余名职工得到妥善安置，支付安置费用1.6亿元。养老保险覆盖面进一步扩大，离退休人员养老金按时足额发放，市属企业下岗保障向失业保险并轨。医疗保险覆盖面80％，解决1万名困难职工医疗保险。城市居民最低生活保障基本实现应保尽保。改善老年大学办学条件，市社会福利院通过国际标准质量管理体系认证。实施了一批惠民工程，改造老城住宅41万平方米，建设经济适用房10万平方米，提供货币配租和廉租房248户，改造便民道路15条，铺装人行道板10万平方米。铺设自来水管道20.4公里、改装户表2.4万户，铺设燃气管道51公里、新增用户7043户。下水道整治、公厕改建、街巷路灯更新等得到有序推进。完成城市绿化360.7公顷，绿化覆盖率和绿地率分别达34.4％和29.5％。

农民生活质量得到提高。农村七件实事成效明显，完成草危房改造10577户，提前一年完成三年改造任务。新增农村改水受益人口38万人。建成农村公路1167公里，新增公交通达村317个。新型农村合作医疗参合率达77.8％。减征农业税1.16亿元，实施粮食直补、良种和农机补贴，农民人均政策性增收54.5元。四级联动帮扶农户53905户，被帮农户人均增收500元。筹集各类扶贫资金1.17亿元，发放扶贫小额贷款5850万元，受益农户1.8万户。投入7900万元实施农业资源综合开发。注重关爱弱势群体，清欠农民工工资1471万元和工程款1.7亿元，救助救济灾民1320万元，免费为千名残疾人配置残疾用具3000多件，老区建设有序推进。建立农村居民最低生活保障制度。完善以敬老院为基础的五保供养体系。及时发放优抚对象和义务兵优待金。义务教育一费制和贫困学生救助工程得到实施。

六、精神文明和民主法制建设不断加强

群众性精神文明创建工作深入开展，省级文明城市创建扎实推进，实施全民创业、奋力赶超主题教育，开展建设学习型城市和与雷锋车同行关爱活动，做好科技、文化、卫生三下乡和科技、文化、法律、卫生四进社区，加强未成年人思想道德建设，社会文明程度和市民文明素质不断提高。

自觉接受法律监督、民主监督、舆论监督和社会广泛监督，定期向人大和政协报告工作、通报情况，主动听取人大代表、政协委员意见和建议，提高建议和提案办理质量，加强领导、狠抓落实、强化督查，较好完成了399件代表建议和委员提案的办理任务。社区居民自治组织建设得到加强，村级重大事务决策、管理和监督不断规范，第七届村委会换届选举进展顺利。四五普法教育广泛开展，全民法律意识和法制观念不断增强。制定法治连云港建设实施纲要，强化社会治安综合治理，开展严打整治斗争，平安连云港建设富有成效。高度重视信访工作，社会矛盾大调解得到加强。加强安全生产基础工作和监督管理，安全生产形势平稳。组织开展农资、食品、药品等执法打假专项活动，大力整治假冒伪劣化肥生产销售，有效开展正源行动保健康等活动，市场经济秩序得到有效整顿和规范。

注重政府自身建设，推进政府职能转变。认真实施《行政许可法》，规范行政行为，扩大政务公开，落实行政执法责任制，依法行政水平逐步提高。推行涉及公众利益重大决策听证制度和专家咨询论证制度，有效提高政府决策民主化科学化水平。推进政府机构改革，深化行政审批制度改革，注重效能建设，强化目标管理，转变工作作风。加强廉政建设，落实廉政建设责任制，强化行政监察和审计监督，开展纠风治乱和行风评议活动，促进了部门和行业风气有效好转。

各位代表，过去一年的工作成绩，是全市人民在中共连云港市委领导下，不断解放思想、开拓进取的结果，是方方面面团结合作、顽强拚搏的结果。在此，我代表市人民政府，向全市广大干部群众、驻连部队、武警官兵、公安干警致以亲切的问候！向各民主党派、工商联、群团组织、各界人士和离退休老同志，向驻连部省属单位，向所有参与和支持连云港建设的海内外朋友表示崇高的敬意！对各位人大代表、政协委员给予政府工作的大力支持表示衷心的感谢！

在肯定成绩的同时，我们清醒地看到，经济和社会发展中还存在一些困难和问题，工作中还有不少薄弱环节：一是经济结构需要进一步优化。一产比重仍然偏高，二三产比重偏低，工业化水平低于全省15个百分点。二是投资发展缺少重大项目支撑。全社会固定资产投资增幅回落较大，新批直接利用外资项目规模低于全省平均水平，带动产业发展的支柱型大项目较少。三是财政收支矛盾仍然突出。虽然财政收入保持较快增长，但可用财力仍然有限，刚性支出大幅度增加，税源经济培育有待加强。四是农民增收长效机机制还没有形成。虽然粮食增产较多、农民收入增长较快，但农业综合生产能力还比较薄弱，加之农资价格涨幅较大，保持农民持续增收还比较困难。五是就业压力和社会保障问题不容忽视。目前还有2万城镇登记失业人员和25万农村常年剩余劳动力需要转移，年度新增就业岗位供不应求。同时，政府职能转变还不适应发展要求，政府工作中既存在少数工作人员违规行政、自律不严的问题，也存在少数部门服务效率不高、行政不作为等现象。我们将高度重视这些问题，在今后的工作中采取有力措施，切实加以解决。

2005年工作任务

2005年是实现十五计划目标的冲刺之年，是人均地区生产总值跨越1000美元台阶、步入新的发展阶段的开局之年。今年政府工作的总体要求是：以邓小平理论和三个代表重要思想为指导，认真贯彻党的十六大、十六届四中全会和省委十届八次全会精神，全面落实科学发展观，按照市委九届五次全会部署，以富民强市、快速崛起，全面建设小康社会总揽全局，全力实施以工兴港、以港兴市、以市带农的发展方针，进一步解放思想、抢抓机遇，进一步深化改革、扩大开放，进一步加快发展、提高质量，坚持统筹兼顾、协调发展，坚持重抓主体、主攻项目，坚持以人为本、富民优先，大力调整经济结构，大力培育特色经济和新的增长点，推进民营经济发展，推进沿东陇海线产业带和东部城区建设，保持全面协调可持续发展的良好势头，建设富强文明、和谐安定的新港城。

2005年国民经济和社会发展的主要预期目标是：地区生产总值增长11％，财政一般预算收入增长13％，全社会固定资产投资增长13％，社会消费品零售总额增长13％，外贸出口增长15％，直接利用外资增长25％，城市居民人均可支配收入增长8％，农民人均纯收入增长7％，城镇登记失业率控制在4.5％以内，人口自然增长率控制在6‰以内，城镇化率提高2个百分点以上。

围绕上述目标和要求，着重抓好以下八个方面工作：

一、不断优化经济结构，壮大经济综合实力

切实做好三农工作，发展繁荣农村经济。推进农业结构调整，在稳定粮食综合生产能力基础上，进一步优化特色优势农产品布局，发展特色乡镇、创建特色县区，做大做强创汇蔬菜、优质瘦肉型猪、高效水产等农业主导产业。围绕建设绿色生态连云港目标，壮大花卉、苗木等园艺业，加快林业生态工程建设和产业化步伐，森林覆盖率提高0.5个百分点。推进海洋和渔业经济快速发展，扩大工厂化养殖和特色渔业规模，建成全省最大的设施渔业和观赏渔业基地，加速发展水产品加工业，以浅海贝类增养殖为重点推进浅海域开发，加强前三岛江苏海珍品养殖基地建设和管理。推动畜牧业加快发展，大力发展食草节粮型畜禽和特色养殖业。建立健全农产品质量标准、检验检测、认证认定体系，完善农产品市场安全准入制度。用发展工业的理念发展农业，推动农业规模化、专业化、集约化、标准化生产，提高农业产业化水平。依托龙头企业配套发展深加工，推动兴云集团、榆城集团等109家规模龙头企业加快发展，重点扶持12家省级龙头企业做大做强。办好江苏国际农洽会，大力推进农业招商，积极发展外向型农业，农产品出口额增长20％。推进农业机械化，推广农业适用技术，发展为农综合服务社，壮大农村经纪人队伍，健全农村服务体系，提高农业综合生产能力。

坚持走有地方特色的新型工业化道路，进一步壮大主体经济。抓住省加快沿东陇海线产业带建设和实施沿海开发的大好机遇，充分发挥差别优势，以临港产业区为核心，以沿陇海线制造业基地、海洋经济开发区、灌河口工业带为支撑，全面释放产业发展势能，着手规划大沿海经济带，构建新兴工业城市产业格局，加快推进临港工业形成高地、三新工业做大做强、传统工业焕发生机、县区工业迅速振兴。推动工业集聚发展，依托沿东陇海线产业带和东部城区，以开发园区为重点，引导工业项目优化布局，新上投资过千万元工业项目85％以上进入园区，力争突破临港大型项目。促进优势产业做强，新医药行业注重吸引战略投资向集团化转变，实现裂变扩张；新材料行业注重推进技术改造和产业配套，拉长产业链条；新能源行业注重核电二期工程及风力发电等项目争取工作，扩大规模效应。引导优强企业做大，加快培育大企业大集团，争取有6户企业营业收入过10亿元，益海粮油销售收入过50亿元。加大工业项目投入，推进企业运用高新技术和先进适用技术改造提升传统产业，实施纺织、化工、机械、食品等一批重点技改项目，完成工业投资150亿元以上。提高质量和效益，定期发布全市质量报告。适应国家宏观调控政策优化投资结构，加快开发特色优势资源，加快发展特色优势产业，加快推动项目达产达效。发挥传统优势进一步培育壮大建筑业，完成增加值29亿元。

大力发展新型服务业，加快构建与城市发展相适应的现代服务体系。围绕提升城市综合服务功能，完善城市商业网点规划，推进服务业对外开放，引进优强企业集团组建物流企业和中介机构，推进市场建设，吸引品牌资源完善现代流通体系，提升商业品位。实现社会消费品零售总额160亿元，居民消费价格指数控制在104左右。整合旅游资源，进一步打造旅游精品，推动重点景区创优升级，提升连云港之夏、西游记文化旅游节品牌效应，挖掘优质资源发展工业旅游、农业生态游、红色旅游。推进旅游度假、休闲、疗养配套发展，加强特色商品开发，发展壮大专业旅行社，拉长旅游产业链，推动旅游业由数量扩张型增长向质量效益型发展转变。推进国民经济信息化，发展壮大信息产业，加快交换网络扩容、基础地理和口岸公共信息平台等项目建设，推进电子政务建设。引导房地产业健康发展，开工房地产240万平方米。普遍推行小区物业管理，鼓励社会力量创办社区便民服务机构。扩大中介服务领域和种类，规范发展律师、会计、审计、评估、行业协会、商会等服务业。用足用好财税政策，支持高新技术产业、民营经济、外向型经济发展，培植壮大财源增长点。充分发挥金融对经济增长的支撑作用，深化银企合作，引导金融机构扩大有效信贷投放，维护金融秩序，促进保险业创新服务。

大力实施以港兴市主战略，进一步激发民营经济活力，加快壮大县域经济实力。全面系统加快港口经济发展，强力推进亿吨大港、现代物流中心建设和临港大开发，努力实现港口功能大提升、临港产业大突破、东部城区大发展，形成港口建设与临港产业互动、城市功能与港口发展配套的联动发展格局。进一步拓展港口岸线，多元投资加快深水大港建设。增加海上航线航班，提高铁路疏运能力，增开五定班列，拓展公路水路疏运，争取开通集装箱绿色通道，完善港口集疏运综合网络，加快建设港口中云物流园区，不断增强以港口为龙头的区域性国际商贸中心城市功能。港口吞吐量突破5000万吨，集装箱运量突破80万标箱、冲刺100万标箱。落实各项扶持政策，完善信用担保体系，激发全民创业潜能，推动民营经济尽快成为全市经济的半壁江山。围绕上规模、上水平，引导民营企业发展三新产业、特色产业、新型服务业，提升产业层次。引导骨干民营企业向园区集聚，参与重点支柱企业的协作配套，促进点状经济向块状经济转变。鼓励民间资本以多种形式进入公益事业、城市建设和社会事业领域。树立强县壮市观念，促进县域经济竞相发展，依托特色优势壮大县域支柱产业，突出发展个性，形成产业特色，强化产业支撑。促进城乡生产要素双向流动，加快城乡经济社会发展协调融合，推进中心城市和县域发展良性互动。通过产业扩张和项目集聚壮大县域综合实力，通过工业化和城镇化做大县域经济规模，在区域争先竞位中实现地区经济的振兴和繁荣。

二、高度关注民生质量，切实改善人民生活

把人民生活摆在突出位置，着力解决关系群众切身利益问题，协调推进城乡就业和社会保障工作，组织实施好市区十项惠民工程和农村十项利民工程，加快推进全面小康社会建设步伐。

实施积极就业政策，推动城乡居民创业就业。积极探索经济与就业双增长的新路子，坚持鼓励创业和推动就业相结合，以创业带动和促进就业。积极发展劳动密集型产业，落实再就业优惠政策，继续实施政府购买岗位等再就业援助工程，新增就业岗位1.8万个。规范劳动力市场秩序和企业用工行为，严格执行最低工资标准，做好破产企业职工安置工作。推进大中专毕业生、复员转业军人就业安置与自主择业。继续实施农村劳务输出百万工程，强化农民技能培训，提高组织化程度，新增劳务输出10万人。严禁拖欠农民工工资，保障农民工合法权益。加快培育和发展城乡统筹的劳动力市场，加强对城镇新成长劳动力和农村富余劳动力的就业指导和服务。

完善社会保障体系，建设稳定祥和城乡环境。在巩固两个确保的基础上，不断完善与经济发展水平相适应的社会保障体系，加大公共财政投入，扩大社会保险覆盖面，将符合条件的城市困难人口全部纳入最低生活保障范围。全面实行农村居民最低生活保障制度，完善农村养老制度，开展农村医疗救助，努力实现农民贫有所济、老有所养、病有所医。切实关注社会弱势群体，健全社会保险、救助、福利和慈善事业相衔接的社会保障体系，对困难群众给予更多的关注和关心，保障失地农民基本生活，搞好残疾人生活救助，建立健全扶困助学机制，保证城乡困难群众及其子女有饭吃、有衣穿、有房住、有学上。

精心组织实施市区十项惠民工程，有效改善居民生活质量。完成老城住宅改造60万平方米，推进住宅平改坡，建设经济适用房20万平方米，提供廉租房350户。新建改建繁荣路、中大街等50条便民道路，基本完成老城区便民道路改造。铺装人行道板20万平方米。整治下水道10公里，解决老城区低洼地段积水问题。新建改建一批公厕，沿街单位厕所对公众开放。全面更新小街巷路灯，消除老城区小街巷无灯现象。新增更新一批公交车辆，改善市民出行条件。铺设自来水管道20公里，改装户表3万户。建成西气东输天然气站和至连云输气管线，铺设燃气管道30公里，新增用户6000户。建设一批城市公园和街心绿地，完成城区绿化面积300公顷，绿化覆盖率和绿地率基本达到国家园林城市标准，创建省级园林城市。

着力组织实施农村十项利民工程，改善农村生产生活条件。农村改水人口覆盖率达95％。建成县乡村道路1000公里，客运班车村通达率达90％。新型农村合作医疗人口覆盖率达85％，启动市郊新型农村合作医疗保险。对符合条件的农村计划生育人口实行每人每月50元奖励扶助制度。完成四级联动致富农民三年帮扶目标。投入8000万元继续推进农业综合开发。多方筹集资金继续加大贫困地区扶贫开发力度。财政支农支出增长高于预算总支出增幅。强化农民负担专项治理和涉农收费公示，取消农业税，有效解决乡村债务和运转经费。围绕建设农村生态家园发展沼气用户5000户。

三、持续推进改革开放，增强经济发展活力

抓住经济发展态势良好、各方面改革意识增强的有利时机，着力推进各项改革，从体制机制上为全面协调可持续发展扫除障碍，以改革创新为动力推动全局工作。进一步深化企业改革，加快工业和商贸流通企业改制，推进工业企业资产重组，盘活存量资产2亿元，完成外贸、粮食、供销、建筑等行业改革。推进企业上市，争取进入上市辅导2家，组建股份有限公司2家，推动条件成熟企业进入发审程序。实施政府机构改革，组建市国资委，履行国有资产出资人职责，整合国有资产经营和管理公司，调整组建发展改革委、食品药品监督管理局、安全生产监督管理局。全面推进事业单位改革，基本完成生产经营型事业单位改制，按照清理、归并、精简、规范的原则，全面推进行政管理型事业单位改革，启动社会公益型事业单位改革。多形式吸收利用社会资本参与公益事业改制、改组和改造，增强公益事业发展活力。继续推进药品生产流通体制改革，完善药品集中招标采购办法，规范药品市场秩序。围绕减人减事减负推进乡镇机构改革。推进民航转制重组。

抓住国内外产业格局加快调整和生产要素加速重组的重大机遇，利用我市特色要素资源日益凸显的差别优势，发挥各类开发园区的载体作用，强力推进新一轮招商引资，在宏观调控新形势下尽快形成项目投资竞相集聚的态势。突破重点区域招商，境外重点加强对日韩、港台招商，充分利用友城、侨务、台商等资源扩大交流与合作，注重研究日韩和台湾地区产业转移趋势和企业投资倾向，力争实现日韩和台湾地区引资倍增目标。境内重点吸引长三角、珠三角民间资本和内陆军工企业，积极承接产业转移。突破特色优势招商，有效组合土地、电力和陆桥运输、深水大港等产业要素，大力推介适宜重大战略性投资和项目布局的产业承接空间，扩大特色优势影响力，形成产业集聚向心力，努力变特色要素优势为产业发展优势。突破重大项目招商，切实加大重大投资项目招引强度，尽快突破一批大项目，在港口建设、临港工业、资源开发、高新产业、特色经济等方面拓展投资空间，确保引办一批过千万美元项目，力争突破超亿美元大项目。突破园区功能招商，充分利用园区载体，按照功能定位有针对性地开展招商引资，国家级开发区重点围绕承接龙头型产业大项目抓紧完善基础条件，县区开发园区重点围绕引办各类成长型项目创出特色、提升档次，加快构建功能配套、布局合理、特色鲜明的园区发展体系，通过提高集聚效应、做大产业规模、强化产业配套、增强产出功能，以点串线推动沿海沿线产业加速扩张。进一步营造亲商安商氛围，尽快建设国家级开发区一站式服务中心、外商生活中心，搞好人性化服务，创建适宜创业、适宜居住的投资环境。

积极开拓国际市场，调整出口商品结构，培育特色出口产品，引导企业扩大本地产品、机电产品和高新技术产品的出口。促进出口生产企业上规模上水平，扩大民营出口经营主体。用足用好出口加工区政策，吸引加工企业进区发展，壮大加工贸易规模，提高加工贸易水平，争取从事加工贸易企业突破100家。加快推进国际商务区建设，逐步完善外贸服务体系，创造良好的外贸环境。鼓励有条件的企业到国外投资办厂、开展工程承包和劳务输出，促进对外经济的快速增长。

四、加快重点项目建设，提升城市带动功能

继续加强基础设施和综合环境建设，抓好水利、交通、电力等30项重点项目。抓住国家实施沂沭泗洪水东调南下工程的有利时机，开工建设新沂河50年一遇工程，实施安峰山等大中型水库除险加固、沭南灌区改造等农村水利工程、东盐河整治等城市防洪工程。续建连盐高速公路，加快国省道网化工程建设，力争开工建设墩尚至临沂高速公路和242省道，改造一批市县际公路，尽快形成中心城区与周边市县联接畅通的干线网络。加快港口基础设施建设，启动15万吨级航道一期工程，基本满足第五代集装箱船全天候通航及10万吨级散货船停泊，推进庙三顺岸工程。做好20万吨级散货码头、液体化工码头等项目前期工作，争取建设集疏港快速通道。建成苍梧等一批220千伏和500千伏输变电工程，建设虎山、龙尾等输变电工程。

围绕创建国家环保模范城市、国家卫生城市、省级文明城市、省级园林城市，切实做好中心城市的精心规划、精致建设和精细管理工作。全面放开规划设计市场，高水平组织修编新海城区出入口、城郊结合部、重点开发地块和主要广场节点规划，精心组织编制东部城区分区规划和港口、开发区专业规划，处理好港口、产业、园区、城市的发展关系，形成分工合理、特色鲜明、功能互补、发展协调的城市新格局。建设市规划展示中心。瞄准现代海滨城市目标，继续坚持经营城市理念，投资50亿元组织实施300项增强城市功能、提升城市形象、改善生态环境、方便群众生活的城市建设项目。集中力量加快东部城区建设，全面推进市政道路、公用设施、土地开发等项目建设，建成新港城大道，撤销朝阳收费站，续建新东方大道，新建滨海路、栖霞路，改建中山东路，建成国际商务中心商务大厦主体工程，强化东部城区功能，提升产业承载能力。继续完善新海城区路网，新建改建南极路、新建东路、解放东路等30条主次干道，畅通一环、二环路网，加快建设三环路网。高起点开发建设凤凰新城；高品位改造龙河广场、新华广场等窗口地段，规划建设一批标志性建筑；高标准改造新海城区南北西三个出入口和东部城区西北两个出入口，刷新城市窗口形象；高水平建设滨海精品工程，开发建设特色海滨观光带。加快东部城区污水处理厂建设，建成大浦垃圾焚烧热电厂主体工程，综合治理市区河道污水。新增人防工程4万平方米，基本建成人防应急指挥中心。推进城市管理由粗放型向精细型、传统管理向依法管理转变，创建20个精细管理样板点，新建亮化精品50项，美化干道楼体15万平方米，有效整治占道经营和脏乱差现象，以点带面提高城市管理整体水平。

坚持城乡统筹发展，搞好城乡协调对接，做大做强四县县城和中心镇。完善四县县城总体规划成果和县域城镇体系规划，加快县城新区开发和工业园区建设，完善市政基础设施，建设四县污水处理厂。发展各具特色的支柱产业和城镇经济，增强对周边地区辐射带动能力。加速构建以中心城市为核心、四县县城为辅翼、小城镇特别是中心镇为基础的梯度结构的现代化城镇新体系，加快城乡一体化进程。

五、大力实施科教兴市，强化发展智力支撑

加快区域性科技创新体系建设。积极推进高新技术产业发展，加快新医药产业基地、东海硅材料产业基地和中电华威、恒瑞医药863基地等高新技术产业基地建设，组织创新药物与中药现代化的工程化、产业化攻关，实施硅材料产业化的共性和关键技术研究。加大技术创新力度，组织实施各类火炬计划项目30项，开发高新技术产品30个，新培育省市高新技术企业8家和制造业信息化示范企业10家。高新技术产业产值增长20％。提升农村特色产业发展档次，重点突破制约支柱产业发展的共性技术，促进产业技术升级。组织实施星火计划50项，农业科技攻关计划10项。进一步加强产学研联合，努力吸引一批大专院校和科研院所建设科研基地，落实一批科技合作项目，推动一批科研成果转化。充分利用与中科院、清华大学等已有的合作关系，在高新产业、区域支柱产业等领域开拓新的协作关系。继续实施百名教授科技兴百村和百名专家科技兴百企工程。加快技术基础设施建设，推进各级工程中心和技术中心发展。推进知识产权创造与保护，加强社会科学和重大战略研究，科学编制国民经济和社会发展第十一个五年计划和2020年远景规划。

围绕办人民满意教育，协调发展各类教育事业。均衡优质发展义务教育，推进义务教育免试就近入学，新扩建中小学校舍10万平方米，小学、初中入学率分别达99.5％和99％。有效实施素质教育，促进教学课程改革，推进信息化和双语教育。加快发展高中段教育，高中段入学率达85％。发挥优质教育资源作用加强普通高中建设，提高东部城区高中教育质量。加快发展中等职业教育特别是农村中等职业教育，重点建设一批示范性骨干学校和专业。着力发展高等教育，支持淮海工学院建设发展，推进连云港师专升本，实施新城区教育事业项目开发。积极发展成人教育和现代远程教育。稳定农村教师队伍，保证教师工资稳步增长。

高度重视人才队伍建设，促进区域人才一体化开发和有序流动。发挥用人单位培养和使用人才的主体作用，大力引进急需的国内外创业型人才，建设一支高技术、高素质的技能人才队伍。建立人才居住证制度，开通人才引进、使用绿色通道，形成创新活跃、流动灵活、各尽其能的人才机制。新增各类人才1万名。

六、加快社会事业进步，促进全面协调发展

发展繁荣文化事业，提高精神产品生产能力，努力创作更多融思想性艺术性观赏性于一体的优秀文艺作品。活跃城乡群众文化，加强社区和农村文化设施建设，深入开展广场文化系列活动，规范文化市场和新闻出版。实施将军崖岩画、孔望山摩崖造像、石棚山摩崖题刻和民族民间文化保护工程。有线电视村联网率达60％，新增用户3万户，积极推行有线数字电视服务。广泛开展全民健身活动，积极发展竞技体育，办好全国十运会沙滩排球、武术散打、帆船帆板等赛事。推进城乡卫生协调发展，完善公共卫生体系，建立疫情报告与公共卫生事件信息网络，加强公益性医疗机构设施建设，强化疾病预防控制和卫生执法监督，传染病发病率低于全省平均水平。加快推进社会事业发展项目建设，建成市少儿活动中心、新博物馆、新闻中心、中医院门诊楼，新建扩建市县传染病医院，开工建设市文化艺术中心、紧急救援中心等项目。增强全民国防意识，加强民兵、预备役部队建设和国防动员工作，做好双拥工作，密切军政军民关系。全面完成经济普查任务，进一步完善统计服务体系。抓好外事、侨务和对台事务，做好档案、气象、地震、民族宗教等工作。

扎实推进可持续发展。做好人口和计划生育工作，提高出生人口素质，稳定低生育水平，计划生育率90％以上。规范计划生育基层基础工作，全面开展关爱女孩行动工程，逐步遏止出生人口性别比升高势头。依法保护和合理利用国土资源，调整优化城乡建设用地，提高土地资源集约利用水平，完善经营性土地使用权招标、拍卖和挂牌出让制度。综合整治开山采石，搞好云台山植被恢复。加强海洋行政管理，注重海洋资源开发与保护，推行海域使用市场化运作。实施生态市建设总体规划，强化城乡环境综合整治，严格控制污染排放，巩固污染源达标排放成果，重视解决跨界水污染问题，确保饮用水源水质达标率保持在98％以上。大力发展循环经济，提高清洁能源使用率。力争通过创建全国环保模范城市和东海、赣榆国家级生态示范区考核验收。

七、加强精神文明建设，营造文明法治环境

坚持用科学发展观引导干部群众，为科学发展、赶超发展营造积极向上的社会氛围和团结奋进的群众基础。以创建省级文明城市为载体，以提高市民文明素质和提升城市文明形象为目标，大力弘扬新时期连云港精神，广泛开展群众性精神文明创建活动，推进城乡共建文化先进村活动，加强未成年人思想道德教育，努力提升城乡整体文明水平，让人民群众充分享受精神文明建设的成果。加强信用体系建设，着力推进政务诚信、商务诚信、社会诚信，全力打造诚信连云港。加强学习型城市建设，努力构建全民学习、终身学习的学习型社会。

大力推进民主政治建设。自觉接受人大及其常委会监督，及时向人大报告工作，落实各项决议、决定，认真执行财政预算。积极支持政协履行政治协商、民主监督、参政议政职能，主动听取各民主党派、工商联及各人民团体的建议和意见。认真办理人大议案、建议和政协提案、建议案，提高办理质量。重视人民群众对政府机关及其工作人员的监督，支持监察、审计部门依法独立履行政府内部监督职责。增强政府工作透明度，建立新闻发言人制度，推进政务、厂务、村务公开，保障人民的知情权和参与权。坚持科学决策、民主决策和依法决策，完善专家咨询论证制度和重大事项社会公示、听证制度。推进基层民主，加强社区居民自治组织建设，整合社区公共资源，改善居委会工作条件，开展安全文明社区创建活动。健全村委会组织建设，开展村民自治示范活动，争取90％的村达到村民自治模范村标准。

全力维护社会安全稳定。实施依法治市五年规划，推进平安连云港和法治连云港建设。落实社会治安综合治理措施，加强治安防控体系建设，严厉打击各种犯罪活动。强化公共安全管理，严密防范和打击邪教、传销等有组织的违法犯罪活动。抓好安全生产基层基础工作，强化安全生产督查考核和责任追究，减少各类事故，遏制重特大事故发生。大力整顿规范市场经济秩序，深入开展农资制假售假和食品药品安全专项整治工作。高度重视信访工作，积极化解人民内部矛盾，妥善处理群体性事件，促进社会和谐稳定、人民安居乐业。

八、强化政府服务功能，建设人民满意政府

面对新形势新任务，我们必须本着一心对人民负责、一切为了人民利益的宗旨，加强政府管理制度、管理方法创新和法规制度建设，努力做到求真务实、开拓创新、勤政高效、清正廉洁。

求真务实推进政府效能建设。树立正确的政绩观，力戒官僚主义、形式主义，把更多的时间精力、财力物力用在推进经济建设、社会事业发展和解决民众实际问题上。增强为市场主体服务意识，创新服务方式，精简审批事项，提高行政效率，强化目标管理，创造良好发展环境。着力提高公务员队伍素质，始终保持干事创业的精神状态，作风务实，讲求高效，抓出实效，干出实绩。

加强学习推进政府开拓创新。坚持解放思想、实事求是、与时俱进，自觉学习和掌握政治、经济、文化、科技、法律等最新知识和理论，树立科学发展观，在经济社会实践中增强判断能力，创新发展思路，提高执政能力，不断创造新的业绩。注重理论与实践结合，善于在变化转折中抢抓机遇，在破难攻坚中开拓创新，在敢闯敢冒中率先突破，开创政府各项工作的新局面。

依法管理推进政府规范行政。认真贯彻《行政许可法》和《行政诉讼法》，进一步增强政府工作人员法治观念，切实增强依法决策意识和依法执政能力，严格按照法定权限和程序行使权力、履行职责，规范行政许可行为。实行执法责任制、过错追究制和行政赔偿制，做到有权必有责、用权受监督、侵权要赔偿、违法受制裁，坚决纠正损害群众利益的一切行为。

从严治政推进政府廉洁从政。切实加强廉政建设和反腐败斗争，坚持从严治政、从严治吏，认真落实党风廉政建设责任制，突出抓好党内监督条例和纪律处分条例的贯彻落实，加大治本力度，认真开展纠风和专项治理，积极构建教育、制度、监督并重的惩治和预防腐败体系。倡导诚信廉明，工作透明，倍加珍惜党和人民的信任，严格遵守廉洁从政准则，保持清正廉明的政治本色。

以人为本推进政府执政为民。政府的一切权力都来自于人民，政府的全部责任都是为人民谋利。牢固确立科学发展观，心中时刻装着人民，干事始终为了群众，把执政为民的要求融入到思想感情里、体现在政策措施中、落实到具体工作上。真正做到权为民所用、情为民所系、利为民所谋，尊重群众、关爱群众、厚待群众，满腔热忱、真心实意地服务于群众。

各位代表，新的一年，任务繁重而艰巨，责任重大而光荣。让我们高举邓小平理论和三个代表重要思想伟大旗帜，紧密团结在以胡锦涛同志为总书记的党中央周围，在中共连云港市委的领导下，与时俱进，开拓创新，励精图治，扎实工作，沿着科学发展轨道奋力赶超，为连云港的繁荣昌盛、人民富裕而努力奋斗！
